# Peri-implantitis increases the risk of medication-related osteonecrosis of the jaws associated with osseointegrated implants in rats treated with zoledronate

**DOI:** 10.1038/s41598-023-49647-4

**Published:** 2024-01-05

**Authors:** Eduardo Quintão Manhanini Souza, Luan Felipe Toro, Vinícius Franzão Ganzaroli, Jéssica de Oliveira Alvarenga Freire, Mariza Akemi Matsumoto, Cláudio Aparecido Casatti, Luciano Tavares Ângelo Cintra, Rogério Leone Buchaim, João Paulo Mardegan Issa, Valdir Gouveia Garcia, Leticia Helena Theodoro, Edilson Ervolino

**Affiliations:** 1 Department of Diagnostic and Surgery, School of Dentistry, São Paulo State University (UNESP), Araçatuba, SP Brazil; 2 Department of Basic Sciences, School of Dentistry, São Paulo State University (UNESP), Araçatuba, SP Brazil; 3 Institute of Biosciences, São Paulo State University (UNESP), Botucatu, SP Brazil; 4 Department of Preventive and Restorative Dentistry, São Paulo State University (UNESP), Araçatuba, SP Brazil; 5 Department of Biological Sciences, Bauru School of Dentistry, University of São Paulo (USP), Bauru, SP Brazil; 6 Department of Basic and Oral Biology, School of Dentistry of Ribeirão Preto, University of São Paulo (USP), Ribeirão Preto, SP Brazil; 7 Latin American Institute of Dental Research and Education (ILAPEO), Curitiba, PR Brazil

**Keywords:** Bone, Risk factors

## Abstract

This study evaluated the peri-implant tissues under normal conditions and under the influence of experimental peri-implantitis (EPI) in osseointegrated implants installed in the maxillae of rats treated with oncologic dosage of zoledronate. Twenty-eight senescent female rats underwent the extraction of the upper incisor and placement of a titanium dental implant (DI). After eight weeks was installated a transmucosal healing screw on DI. After nine weeks, the following groups were formed: VEH, ZOL, VEH-EPI and ZOL-EPI. From the 9th until the 19th, VEH and VEH-EPI groups received vehicle and ZOL and ZOL-EPI groups received zoledronate. At the 14th week, a cotton ligature was installed around the DI in VEH-EPI and ZOL-EPI groups to induce the EPI. At the 19th week, euthanasia was performed, and the maxillae were processed so that at the implanted sites were analyzed: histological aspects and the percentage of total bone tissue (PTBT) and non-vital bone tissue (PNVBT), along with TNFα, IL-1β, VEGF, OCN and TRAP immunolabeling. ZOL group presented mild persistent peri-implant inflammation, higher PNVBT and TNFα and IL-1β immunolabeling, but lower for VEGF, OCN and TRAP in comparison with VEH group. ZOL-EPI group exhibited exuberant peri-implant inflammation, higher PNVBT and TNFα and IL-1β immunolabeling when compared with ZOL and VEH-EPI groups. Zoledronate disrupted peri-implant environment, causing mild persistent inflammation and increasing the quantity of non-vital bone tissue. Besides, associated with the EPI there were an exacerbated inflammation and even greater increase in the quantity of non-vital bone around the DI, which makes this condition a risk factor for medication-related osteonecrosis of the jaws.

## Introduction

Medication-related osteonecrosis of the jaws (MRONJ) is an adverse effect predominantly caused by the use of potent antiresorptive drugs as the bisphosphonates (BP) and the receptor activator of nuclear kappa B factor ligand inhibitor (RANKL), denosumab^1^. MRONJ is characterized by the presence of exposed bone (or that can be probed throughout intra or extra oral fistulae) in the maxillofacial region, and that persists with no healing signs after eight weeks of onset, in patients with previous or current treatment using antiresorptive and/or antiangiogenic drugs, with no previous history of radiotherapy or metastatic disease in the jaws^1^.

The ethiopathology of MRONJ is not fully elucidated yet; however, studies point to a highly complex multifactorial nature. Nowadays, there are some etiopathogenic factors related to MRONJ development and onset caused by bisphosphonates as the inhibition of resorptive activity of osteoclasts, which may play a central role in this disease, associated with local inflammation and/or infection, antiangiogenic effect, cytotoxic effect on soft tissue, along with a dysfunction of innate and acquired immunological response^2–4^. The limited comprehension of MRONJ etiology makes treatment difficult and time-consuming, causing severe consequences in the patient. Thus, MRONJ prevention is the ideal strategy^5^.

Among drug-related risk factors, the higher the antiresorptive potency, dosage and treatment duration, the higher is the risk of developing MRONJ. Besides, the use of these drugs in association with corticoids and chemotherapeutic agents can potentialize these risks^6^. Considering the factors related to the patient, those of greatest concern are aging, sex, and comorbidities as cancer, diabetes, and obesity, while dento-alveolar surgeries is among the main local factors (62–82% of MRONJ cases), especially when there is an infectious inflammatory condition associated to the affected tooth^1^.

MRONJ related to dental implants (DI) have been increasingly reported in the last years. Clinical investigations have considered it as an early complication in about 1/3 of the patients, usually during the first six months after implant installation, when peri-implant healing process is underway^7,8^. It can also present as a late complication in around 2/3 of the patients, and its development can be related with an already osseointegrated implant if it occurs after six months of DI installation, that means when peri-implant healing is concluded^7–18^.

In order to prevent MRONJ-DI, the installation of DI in individuals under administration of oncologic doses of antiresorptive drugs has been contra-indicated. Differently, it is not contra-indicated for those patients under osteoporotic dosage^19,20^. However, a matter of concern is focused on those individuals that are already rehabilitated with osseointegrated DI and that, in some point in life, need to be treated with antiresorptive drugs^19,20^. These patients comprise most of the cases of MRONJ-DI and there is a tendency that this become more frequent, since rehabilitation through DI has become popular in recent decades, in addition, life expectancy of the global population is increasing and the use of drugs for the treatment of skeleton-related diseases has been more and more frequent.

Another worrying finding is a supposed correlation between the peri-implantitis (PI) and MRONJ-DI^7,21–24^. PI is a pathological condition primarily caused by bacteria but strongly influenced by local and systemic factors that affects peri-implant tissue, characterized by local inflammation and progressive bone loss that can lead to implant loss^25^. It can affect up to 57% of the patients and up to 28% of the DIs, representing the most common cause of late DI loss^26–28^. Thus, the treatment with antiresorptive medication can contribute with DI loss, and also be considered a triggering factor for MRONJ-DI.

The alterations that occur at cellular and tissue levels around the osseointegrated DI during or after antiresorptive drugs treatment can help elucidate the pathophysiological mechanisms involved in MRONJ-DI, and consequently shed light on prevention and treatment. The aim of this study was to evaluate the peri-implant tissues under normal conditions and under the influence of experimental peri-implantitis (EPI) induced by ligature, in osseointegrated dental implants in the maxillae of senescent female rats previously treated with oncologic dosage of zoledronate.

## Results

### General health status and intraoral clinical examination

In general, the animals were healthy and stable during all the experimental period, well tolerating the surgical procedures and the drug protocols with vehicle or zoledronate. Individually, all the animals presented higher body weight previously to DI installation when compared to the reopening; however, the animals satisfactory responded to the surgical procedure and to the triturated diet, gaining weight throughout the experiment.

From intra-group analysis, none of the groups presented statistical differences in the body weight considering the beginning of the experiment, implant reopening, and euthanasia. From inter-group observation, statistical results did not reveal significant differences related to the body weigh in the three analyzed periods, independent of the different treatments.

No clinical differences were observed in the intra-oral examination and visual inspection between VEH and ZOL groups throughout the experiment. The DI were stable, and the soft tissues were healed; however, ZOL group presented a slight pronounced erythema around the implants in comparison to VEH group. Animals from VEH-EPI and ZOL-EPI groups exhibited large accumulation of biofilm around the cotton ligature at the moment of the samples collection, when tissue erythema and swelling was noted around the implants, more pronounced in ZOL-EPI group. The induction of EPI in VEH-EPI and ZOL-EPI groups did not cause the loss of implant stability in none of the animals. Also, no group presented bone exposure close to the implants at clinical examination.

### Histological aspects of peri-implant tissues

VEH group exhibited a high vascularized dense irregular connective tissue around the implants, showing numerous fibroblasts, and a few leukocytes. In this group, no peri-implant bone loss or active bone resorption were observed close to alveolar bone crest. Bone tissue around the implant presented preserved structure and cell pattern. VEH-EPI group showed connective tissue infiltrated by leukocytes, predominantly, surrounding the implant. Active bone loss was observed in the cervical portion of the implants, considering the presence of osteoclasts resorbing the bone close to the alveolar crest. Only a few areas around the implants presented non-vital bone (Supplementary Materials 1, 2).

In ZOL group, a concentration of leukocytes in the peri-implant connective tissue was observed, predominantly mononuclear cells. In this group, no bone loss was detected. The amount of bone tissue around the implant was similar to VEH group; however, de amount of non-vital bone was significantly higher. ZOL-EPI group showed intense peri-implant mononuclear inflammation, which was more exacerbated than the other groups. A discreet bone loss was noted, which was limited to the cervical portion of the implant. Some large, rounded, and hypernucleated osteoclasts were observed close the alveolar bone crest. Nonetheless, most of them were distant from bone surface, a morphological aspect of inactivity. Most of the specimens of this group presented compromised bone vitality around the implants, although de extension of non-vital bone tissue has been variable among the specimens of this group (Supplementary Materials 1, 2).

### PTBT and PNVBT around the DI

VEH-EPI group (51.8 ± 6.9) presented lower PTBT when compared with the other groups. No statistical difference was detected between VEH (67.8 ± 6.8), ZOL (75.0 ± 8.9) and ZOL-EPI (65.1 ± 5.3) groups.

**Figure 1:**
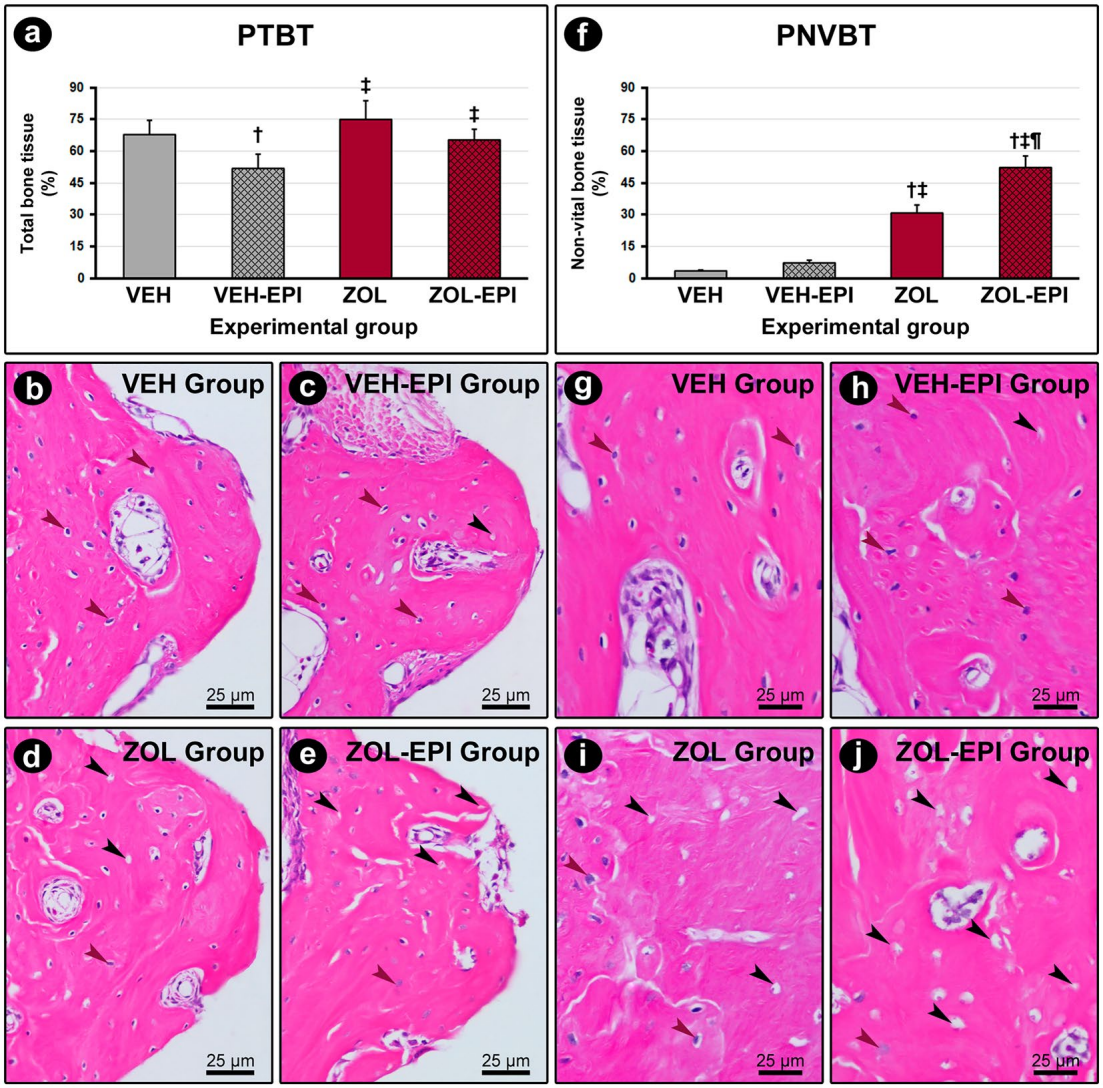
Percentage of total bone tissue (PTBT) and percentage of non-vital bone tissue (PNVBT) in peri-implant tissue. (**a**) and (**f**) Graphs showing PTBT (**a**) and PNVBT (**f**). (**b**–**e**) and (**g**–**j**) Photomicrographs showing the pattern of structure and cellularity of the bone tissue located in the implant threads (**b**–**e**) and in the immediate vicinity of the threads (**g–j**) in groups VEH ((**b**) and (**g**)), VEH-EPI ((**c**) and (**h**)), ZOL ((**d**) and (**i**)) and ZOL-EPI ((**e**) and (**j**)). Statistical test: Variance Analysis (ANOVA) followed by Tukey post-test. Symbols: red arrows, osteocytes; black arrows, empty lacuna; †statistically significant difference in relation to the VEH group; ‡statistically significant difference in relation to the VEH-EPI group; ¶statistically significant difference in relation to the ZOL group. Original magnification: 1000 × . Scale bars: 25 μm.

Considering PNVBT, no statistical difference was observed in the comparison between VEH (3.3 ± 0.7) and VEH-EPI (7.2 ± 1.3) groups. On the other side, ZOL group (30.6 ± 4.1) reveled higher PNVBT when compared with VEH and VEH-EPI groups. ZOL-EPI group (52.1 ± 5.8) showed higher PNVBT in the comparison between the other groups. PTBT and PNVBT in the different experimental groups are presented in Fig. 1.

### TRAP in peri-implant tissues

**Figure 2:**
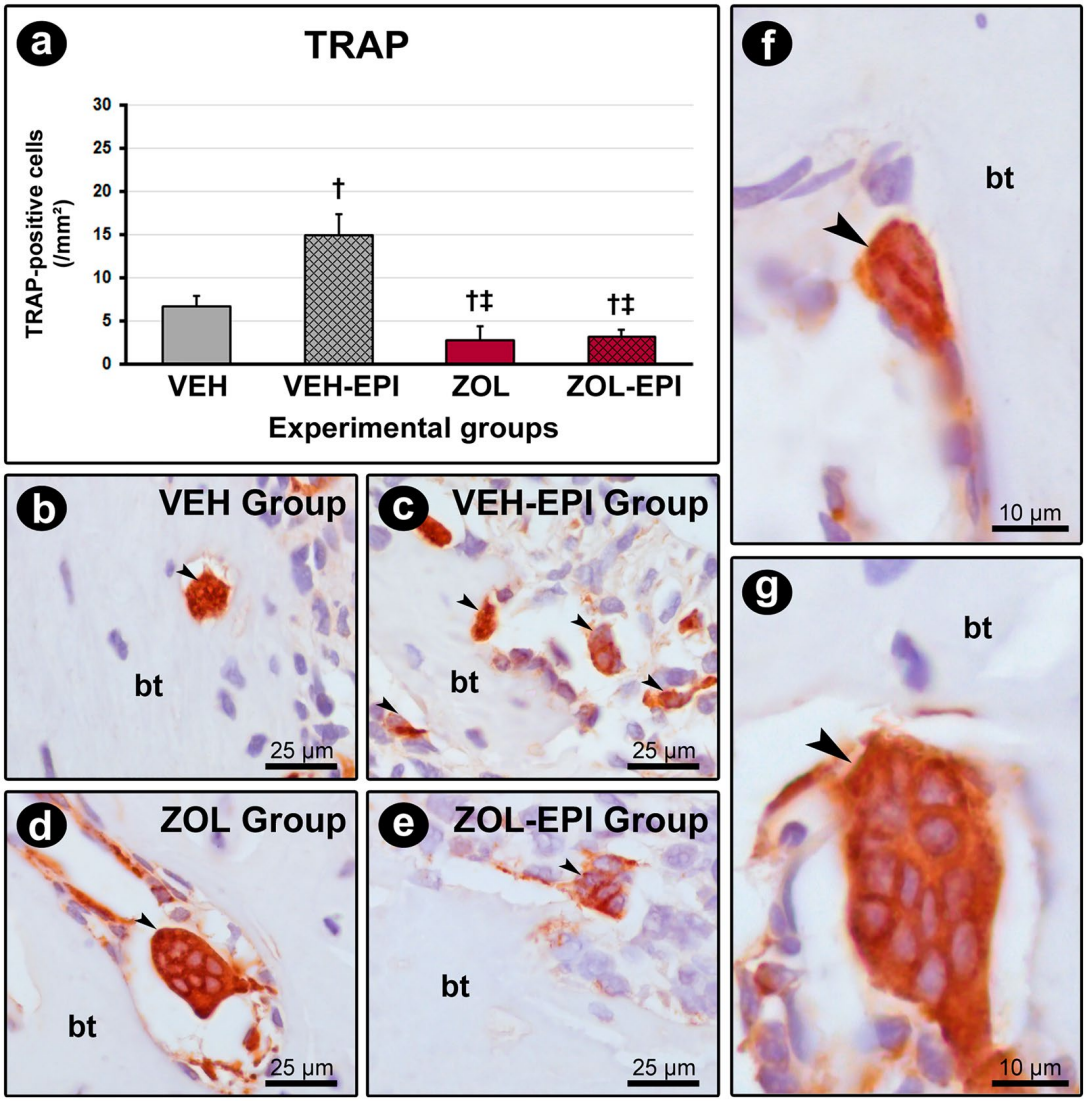
Immunolabeling for TRAP in peri-implant bone tissue. (**a**) Graph showing the number of TRAP-positive cells/mm^2^ of bone tissue. (**b**–**e**) Photomicrographs showing the immunolabeling pattern for TRAP in the peri-implant bone tissue in groups VEH (**b**), VEH-EPI (**c**), ZOL (**d**) and ZOL-EPI (**e**). (**f**–**g**) Photomicrographs highlighting the differences between osteoclasts from the vehicle-treated and zoledronate-treated groups, in which such cells appear much larger, hypernucleated, rounded and distant from the bone matrix. Statistical test used: Variance Analysis (ANOVA) followed by Tukey post-test. Symbols: black arrows, TRAP-positive cells (osteoclasts); †statistically significant difference in relation to the VEH group; ‡statistically significant difference in relation to the VEH-EPI group. Counterstain: Harris Hematoxylin. Original magnification: (**b**–**e**) 1000 × ; (**f**–**g**) 4000 × . Scale bars: (**b**–**e**) 25 μm; (**f**–**g**) 10 µm.

VEH-EPI group (15.0 ± 2.4) showed higher number of TRAP-positive cells when compared with VEH (6.7 ± 1.2), ZOL (2.8 ± 1.7) and ZOL-EPI (3.2 ± 0.8) groups. The groups treated with the zoledronate (ZOL and ZOL-EPI groups) presented lower number of TRAP-positive cells in the comparison with the groups treated with the vehicle (VEH and VEH-EPI groups). Quantification of TRAP-positive cells in the different experimental groups is presented in Fig. 2.

### TNFα, IL-1β, VEGF and OCN in peri-implant tissues

**Figure 3:**
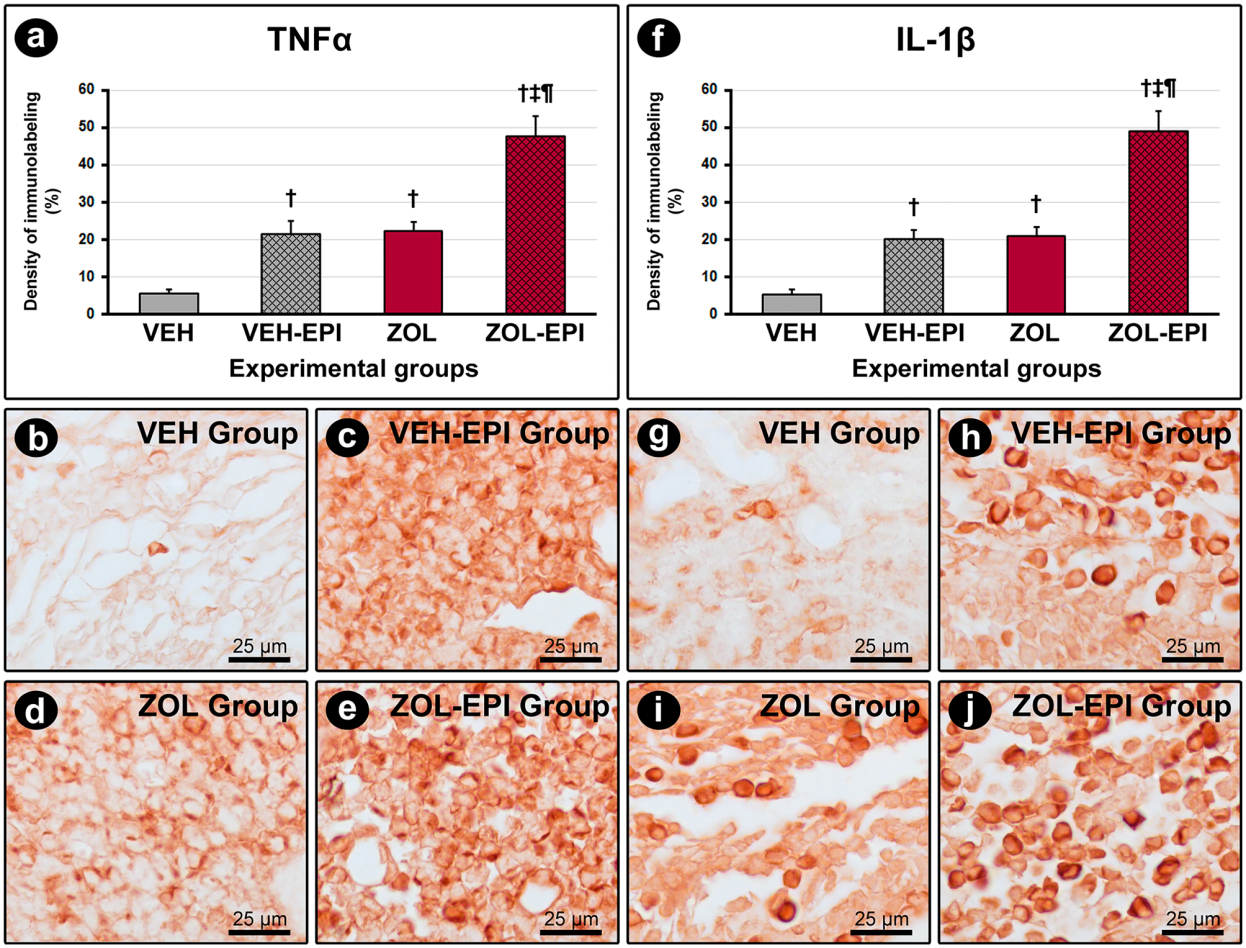
Immunolabeling for TNFα and IL-1β in peri-implant tissue. (**a**) and (**f**) Graphs showing the immunolabeling density for TNFα (**a**) and IL-1β (**f**). (**b**–**e**) and (**g**–**j**) Photomicrographs showing the immunolabeling pattern for TNFα (**b**–**e**) and IL-1β (**g**–**j**) in the peri-implant tissue in groups VEH ((**b**) and (**g**)), VEH-EPI (c and h), ZOL ((**d**) and (**i**)) and ZOL-EPI ((**e**) and (**j**)). Statistical test: Variance Analysis (ANOVA) followed by Tukey post-test. Symbols: †statistically significant difference in relation to the VEH group; ‡statistically significant difference in relation to the VEH-EPI group; ¶statistically significant difference in relation to the ZOL group. Original magnification: 1000x. Scale bars: 25 μm.

VEH-EPI (21.4 ± 3.6, TNFα; 20.0 ± 2.4, IL-1β) and ZOL (22.1 ± 2.5, TNFα; 20.8 ± 2.6, IL-1β) groups presented higher immunolabeling density for TNFα and IL-1β when compared with VEH group (5.4 ± 1.0, TNFα; 5.2 ± 1.3, IL-1β). These targets were also higher in ZOL-EPI group (47.7 ± 5.2, TNFα; 48.9 ± 5.3, IL-1β) in comparison with the other groups. The density of TNFα and IL-1β immunolabeling in the different groups is represented in Fig. 3.

**Figure 4:**
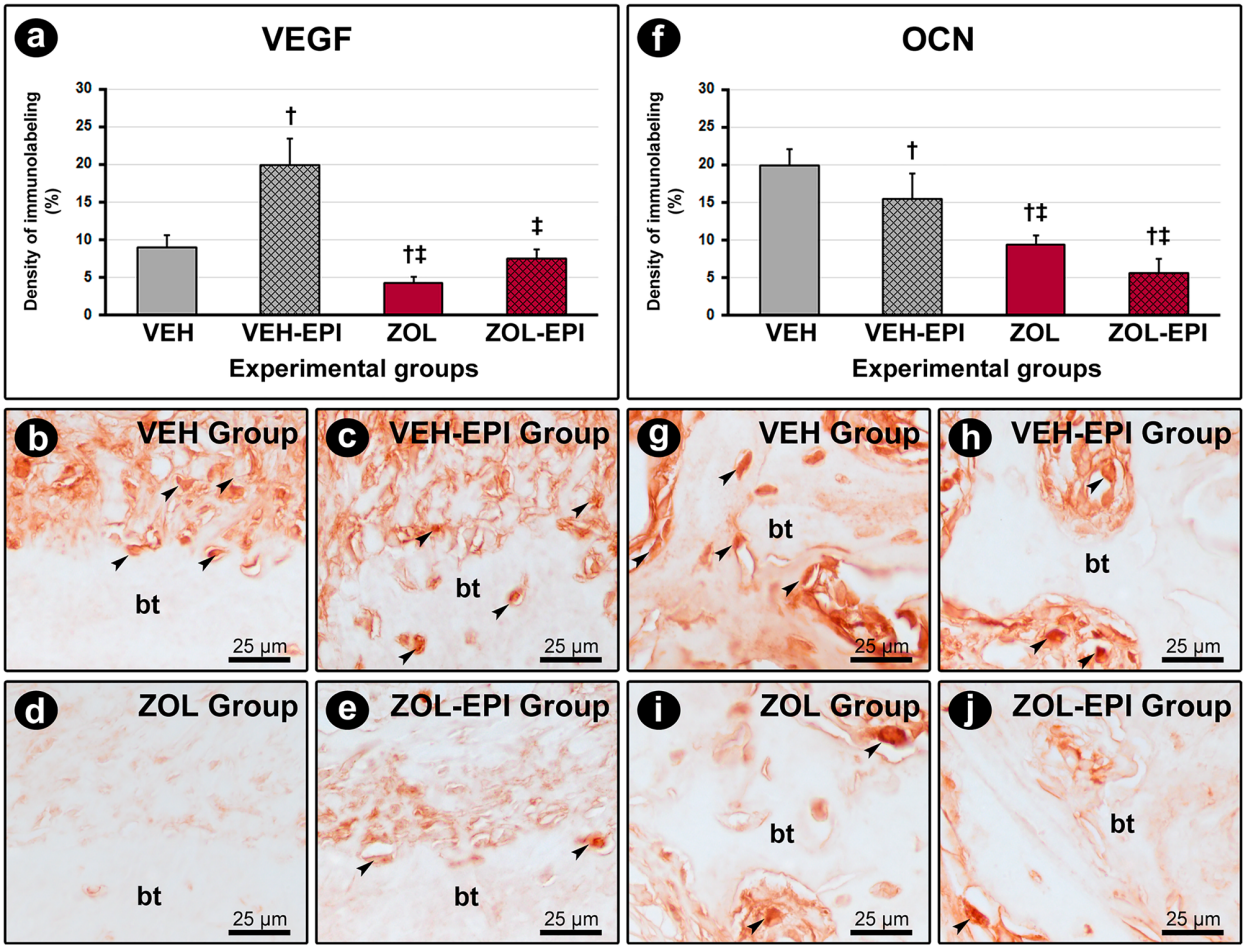
Immunolabeling for VEGF and OCN in peri-implant tissue. (**a**) and (**f**) Graphs showing the immunolabeling density for VEGF (**a**) and OCN (**f**). (**b**–**e**) and (**g**–**j**) Photomicrographs showing the immunolabeling pattern for VEGF (**b**–**e**) and OCN (**g**–**j**) in the peri-implant tissue in groups VEH ((**b**) and (**g**)), VEH-EPI (c and h), ZOL ((**d**) and (**i**)) and ZOL-EPI ((**e**) and (**j**)). Statistical test: Variance Analysis (ANOVA) followed by Tukey post-test. Symbols: †statistically significant difference in relation to the VEH group; ‡statistically significant difference in relation to the VEH-EPI group; ¶statistically significant difference in relation to the ZOL group. Original magnification: 1000x. Scale bars: 25 μm.

VEGF immunolabeling was higher in VEH-EPI group (19.9 ± 3.5) when compared with VEH (8.9 ± 1.7), ZOL (4.2 ± 0.8) and ZOL-EPI (7.4 ± 1.2) groups. In ZOL group VEGF immunolabeling was lower in relation with VEH group. OCN immunolabeling was higher in VEH group (19.9 ± 2.2) in relation with VEH-EPI (15.4 ± 3.4), ZOL (9.3 ± 1.3) and ZOL-EPI (5.6 ± 1.8) groups. As for ZOL and ZOL-EPI groups, OCN immunolabeling was lower when compared with VEH-EPI group. VEGF and OCN immunolabeling in the different groups are presented in Fig. 4.

## Discussion

The present study investigated changes at the cellular and tissue level around DI that were already osseointegrated, which could answer whether their presence could be related to MRONJ during treatment with high dosage of zoledronate. In addition, it was also investigated whether the EPI would be able to generate alterations at the cellular and tissue level in the vicinity of the already osseointegrated DI that could be related to MRONJ during a treatment with oncologic dosage of zoledronate.

Experimental models of MRONJ in animals have contributed with the understanding about its etiopathogenesis^29^, in addition, they have contributed with the proposing and evaluating preventive and curative treatments. With the purpose of exploring the relation among MRONJ, DI and EPI, some methodological challenges had to be surpassed in this investigation. First, only a few experimental models, especially with rats, investigate DI installed in maxillae or mandible. Amongst them, most analyzed socket healing in the maxillae after the extraction of the first upper molar^3,30–32^ or the edentulous space anterior to the molars^33,34^ that demand the use of a short implant, not to involve the maxillary sinus. Another important aspect is about the animal models of EPI. EPI can be achieved by the injection of bacteria^30,34^, lipopolysaccharides (LPS)^31,35^, or using cotton ligatures around the implant^32,33,36,37^. Cotton ligature is very interesting, since it stimulates the orchestrated formation of a multimicrobial biofilm that initiate inflammation and destructive peri-implant response, mimicking what happens in human being. The use of a very short implant associated with the ligature can induce bone destruction that leads to implant loss in a short time. In the present study, if the time of progression of EPI was reduced in order to preserve DI, probably there would be not enough time to develop an adaptative response at the cellular and tissue level around DI.

That is why we present a new experimental model using a longer implant, with 5.7 mm length and 2.5 mm of diameter, immediately installed in the post-extracted socket of the upper incisor of the rats. After osseointegration period, implant platform was exposed and a transmucosal healing screw was connected to the implant, and in VEH-EPI and ZOL-EPI groups the ligature was placed, remaining for five weeks, time enough for the effects of the antiresorptive drug and EPI would occur and cell and tissue reactions around DI could be observed.

Importantly, the new experimental model was based on epidemiologic data about MRONJ, and we tried to mimic the main risk factors and conditions related to it. We used senescent female rats, which were 20-months old at the end of the experiment, considering that MRONJ affects aged women more frequently^1,38,39^, and that aging is also an aggravating factor related with peri-implantitis development^40^. The antiresorptive drug used in this study was the zoledronate in oncologic dosage, since most of the MRONJ-DI is related predominantly with this bisphosphonate^8,9,12,15–17^, frequently used as adjuvant for cancer therapy. Besides investigating the changes that the DI causes in peri-implant tissues, we also introduced a local worsening factor that was the EPI, also related to MRONJ-DI^7,21–24^. In the present study, no implant loss was caused by treatment with zoledronate. Also, no clinical manifestation of MRONJ-DI comparable to humans was observed. Histopathological features seen in ZOL-EPI group can be correspondent to stage 0 of MRONJ or it can be considered as the manifestation of MRONJ-DI in this specific animal model.

Bisphosphonates containing nitrogen, as the zoledronate, inhibits the key enzyme of mevalonate pathway farnesyl diphosphate synthase, which in turn, blocked the generation of farnesyl diphosphate and geranylgeranyl diphosphate, essential for the prenylation of small GTPases. These are crucial for a number of osteoclasts activities as the inhibition of premature apoptosis, morphological changes, organization of cytoskeleton and vesicles traffic, fundamental for bone resorption^41^. From our results it was observed that zoledronate affected the osteoclasts in the tissues around the DI, once TRAP-positive cells were decreased in ZOL and ZOL-EPI groups in comparison with the animals treated with the vehicle. Even the presence of EPI in the animals under zoledronate treatment was not able to significantly increase TRAP-positive cells, resulting in a small peri-implant bone loss in this group, observed in histopathological analysis. Interestingly, the osteoclasts in the animals treated with zoledronate were large, round-shaped, hypernucleated and frequently not attached to the bone surface. On the other side, VEH group revealed few TRAP-positive cells, probably related to osteoclasts involved in local bone remodeling. The EPI in the vehicle treated animals increased TRAP-positive cells with morphological aspect of active osteoclasts, coherent with the peri-implant bone loss observed in histopathological analysis, which validated our peri-implantitis model.

Kwon et al*.*^7^ described three different aspects of MRONJ-DI: (1) frozen type—large area occupied by necrotic bone presenting lacunae with no osteocytes, and bone edges with different degrees of inflammatory cells; (2) osteolytic type—similar to a conventional osteomyelitis, presenting fragments of necrotic bone surrounded by a layer of leukocytes and bacterial colonies, and (3) en bloc type—bone sequestrum involving the implant with empty lacunae, inflammatory infiltrate and biofilm in bone cavities, without losing bone-implant contact. However, the three types can co-exist in the same lesion, depending on the bone destruction and infection severity^7^. Establishing a correlation with the present study, although we did not have the clinical manifestation of MRONJ-DI in the way that it is observed in humans, we verified, analyzing the histological aspect of the peri-implant tissues, that most of the specimens from the ZOL-EPI group showed areas of non-vital bone tissue around the implant and the presence of intense inflammatory infiltrate, and in some specimens, large bone sequestrations, that is, resembling the previously described frozen and osteolitic types, which we believe is a characteristic of the present experimental model.

Amongst the histological parameters evaluated in this study, two of them are fundamental in order to characterize a MRONJ-DI like lesion: the intensity of tissue inflammation and PNVBT. Histopathology and immunohistochemistry for TNFα e IL-1β revealed a mild and persistent inflammation around the DI in ZOL group, but intense inflammation in ZOL-EPI group. Besides, both histopathology and histometry showed increased non-vital bone around the DI in ZOL group, which was exacerbated when EPI was associated (ZOL-EPI group). It seems that there is a strong relation between the intensity and permanence of inflammation and the increased amount of non-vital bone. Probably, the clinical manifestation of MRONJ-DI would happen when a threshold amount of non-vital bone tissue present around the implant is exceeded.

The increase of pro-inflammatory cytokines as TNFα, IL1-β, IL-18 and IL-6, is strongly related with MRONJ in experimental models^42–45^. Morita et al*.*^43^ reported that knockout mice for TNFα, IL-1α/β and IL16 are resistant to the development of MRONJ-like lesions, even when medicated with high doses of bisphosphonates. Drugs like the TNFα inhibitor Etanercepte, and neutralizing antibodies for IL-6, have been suggested as potential drugs that are able to prevent MRONJ in rats treated with zoledronate and submitted to exodontia^45^.

High levels of local or systemic inflammation associated with the inhibition of osteoclast activity induced by zoledronate, stimulate the conversion of pre-osteoclasts into macrophages, that in turn, secret more inflammatory cytokines until an “inflammatory storm” is created^43,45^. Zoledronate has been considered an important modulator of macrophages phenotype, both in vitro and in vivo^46^, increasing M1 polarization and, consequently, activating the pro-inflammatory transcription factor NF-κB (nuclear factor kappa B), and suppressing M2 polarization related to anti-inflammatory events^47^.

In bone tissue, probably the association of the inflammatory events surpasses the capacity of physiological adaptation of the osteocytes, leading them to death via apoptosis, autophagia, necroptosis or necrosis. TNFα/TNFR1signaling has been strongly related with the activation of osteocytes necroptosis, a type of programmed cell death caused by the cell membrane disruption and the release of damaged-associated molecular patterns (DAMPS) in the extracellular matrix. The presence of antigen-associated molecular patterns (PAMPS), LPS, damaged DNAs, amongst several molecules, activate some ligands, as the Toll-like receptors 2/4 (TLR 2/4) that also trigger the necroptosis of the osteocytes, causing secondary inflammatory events that increase the installed inflammation, once these cells are trapped in bone matrix, impeding the subsequent action of the osteoclasts^48^.

The effects of zoledronate on the osteoblasts also deserve attention. ZOL and ZOL-EPI groups revealed significant reduction in immunolabeling for OCN in comparison with the other groups. This might have happened due the cytotoxic effects of zoledronate on the osteoblastic cells’ lineage, or, because of the damages caused by local exacerbated inflammation. Huang et al*.*^49^ reported in vitro the dose-dependent inhibitory effects for type I collagen, alkaline phosphatase and OCN of osteoblasts treated with zoledronate, along with a decrease in the differentiation of precursor cells because of the reduced expression of bone morphogenetic protein 2 (BMP-2). Cytotoxic effects related to proliferation, maturing, and protein expression changes in osteoblast lineage were also point of attention of previous study using zoledronate^50^.

The influence of the bisphosphonates on the vasculature is also considered a negative local factor. In vitro studies revealed a harmful effect of the bisphosphonates, especially zoledronate, on endothelial cells and their precursors, and in vivo investigations related that it severely impairs angiogenesis^51–53^. In this study, immunolabeling for VEGF, one of the key regulators of angiogenesis, was lower in ZOL and ZOL-EPI groups. Previous study from our group also showed that animals that underwent exodontia under zoledronate treatment presented reduced immunolabeling for VEGF when compared with controls^54^. These findings corroborate clinical studies in humans that showed a reduction in serum levels of VEGF during the treatment with both osteoporotic and oncologic dosage of zoledronate^55^.

Although the findings of the present study alone should be seen with concern, another aspect that deserves great attention, and which constitutes one of the limitations of the experimental model employed, is related to the load on such implants, which is minimum in this study. Studies using animals and human samples reveal that the accumulation of non-repaired microcracks in bone tissue can be involved in MRONJ pathogenesis^56,57^. In this way, when the functional load is on these implants, more negative effects can occur in the peri-implant tissues, as the non-repaired microcracks.

Although in the present study a microbiological evaluation was not performed, this aspect should also be considered. Until now, it has not been proven whether microorganisms play a primary or secondary role in the pathogenesis of MRONJ, however, it is proven that they greatly aggravate this condition. Some studies have pointed to the involvement of Actinomyces spp in MRONJ^58,59^. Other more comprehensive studies have shown alterations in Porphyromonas, Lactobacillus, Tannerella, Prevotella, Actinomyces, Treponema, Streptococcus and Fusobacterium^60,61^, that is, osteonecrotic lesions present bacteria representative of the periodontal microflora. It cannot be ignored that some bacteria associated with peri-implantitis are also recognized as periodontopathogens^62–64^, among them P. gingivalis, may also be relevant in MRONJ pathogenesis^65^.

MRONJ severely compromises the patients’ quality of life, especially those that are under therapy for concomitant diseases as cancer^66,67^, since the treatment is usually of long-term, with uncertain prognosis, and can result in severe sequelae. In the case of MRONJ-DI, in addition to implant loss, neighboring teeth or implants may also be compromised. The removal of the DI and necrotic bone may not refrain the progress of osteonecrosis, resulting in large bone losses in maxilla or mandible, making difficult or even impeding a future oral rehabilitation, resulting in further negative consequences on the patient's quality of life. Preventive strategies are ideal when it comes to MRONJ, including MRONJ-DI. We believe that monitoring patients with implants and undergoing treatment, or even after treatment with anti-resorptive drugs, is of fundamental importance. In this case, regular visits to the dentist are essential for hygiene instruction and assessment, as well as maintenance therapies for peri-implant prevention. Controlled and randomized clinical studies, proposing and evaluating protocols to be used in patients with implants and who are, or have been under treatment with drugs with anti-resorptive action, have not yet been carried out and are extremely necessary.

It is concluded that treatment with zoledronate causes changes at the peri-implant level, as a mild but persistent inflammation and an increase in non-vital bone tissue around DI. Furthermore, in the presence of peri-implantitis, there is a significant exacerbation of inflammation and an even greater increase in the amount of non-vital bone tissue, which places this condition as an important local risk factor for MRONJ-DI. Therefore, the sites where DI is installed require strict periodic monitoring during and after treatment with anti-resorptive drugs so that peri-implantitis is avoided.

## Methods

### Animals and randomization

Twenty-eight female Wistar rats (*Rattus norvergicus*), 20-months old, and mean weigh of 400 g were used. The animals were obtained from Central Animal Facilities of State of São Paulo State University, School of Dentistry (FOA-UNESP), Brazil, and were maintained under the following conditions: 12 h-12 h dark–light cycle, room temperature of 22 ± 2 °C, with ventilation and exhaust system allowing 20 air changes per hour, relative humidity of 55 ± 5%. The animals were maintained in plastic cages with a maximum of 4 rats per cage, with free access to water and food. Experimental procedures were performed in accordance with ARRIVE guidelines (https://arriveguidelines.org). Manipulation and experimental procedures were also performed according to the Brazilian National Counseling of Animal Experimentation Control (CONCEA) and the experimental protocol was approved by the Institutional Ethics Committee of Animal Use (#01,006–2018 FOA-UNESP, Araçatuba—SP, Brazil).

The present study followed a controlled, randomized and blind design. Each animal was identified with a numerical sequence from 1 to 28. Minitab^®^ 17 software (Minitab Inc., State College, PA, USA) was used to generate a table containing the randomized distribution of numbered animals in the different experimental groups.

### Upper right incisor extraction and DI installation

**Figure 5:**
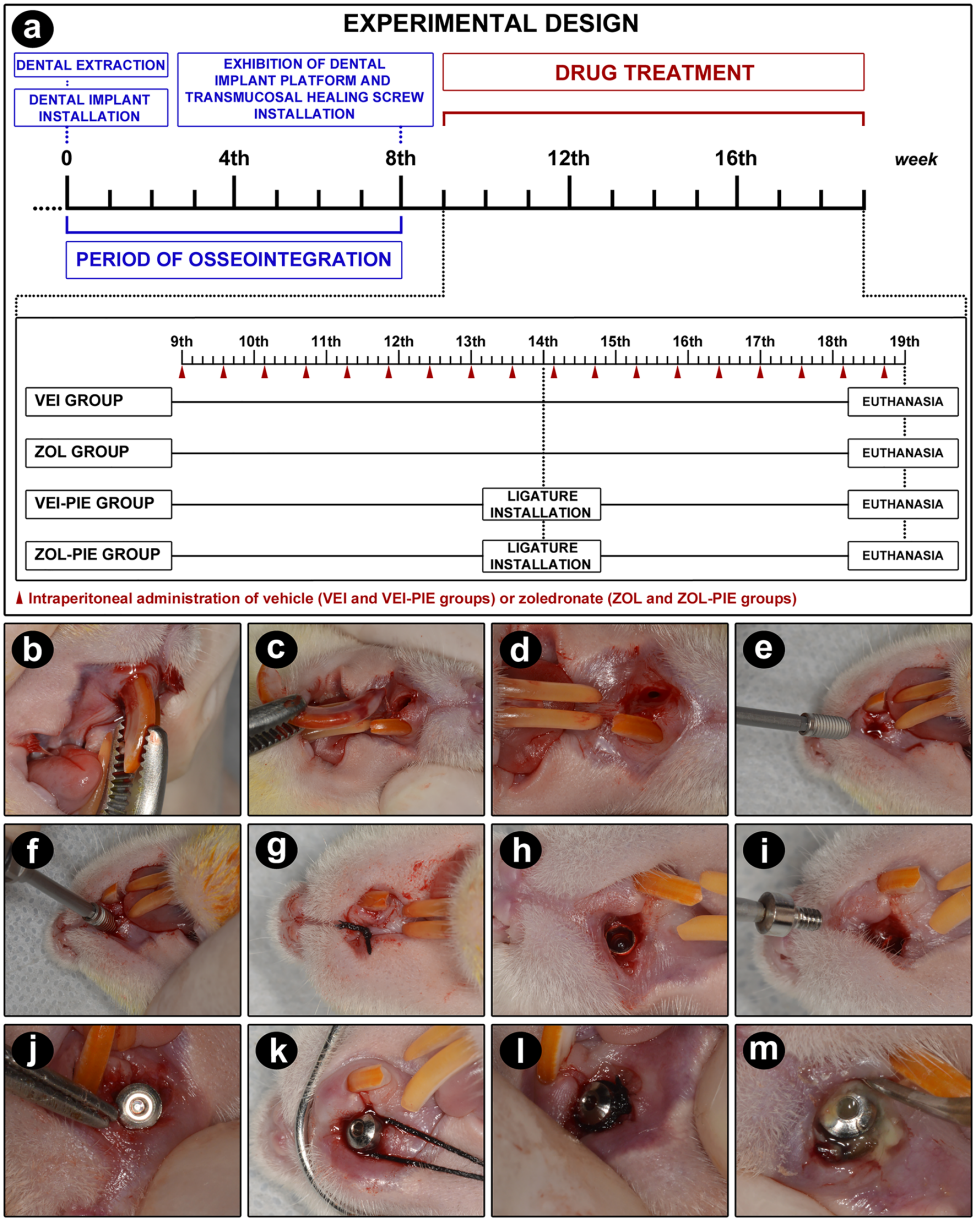
Experimental design. (**a**) Scheme illustrating the experimental design of the study and the main procedures performed over time in each experimental group. (**b**–**d**) Extraction of the upper incisor and clinical aspect of the dental socket after extraction, prior to installation of the titanium dental implant. (**e**–**g**) Dental implant being installed in the dental socket with the aid of the digital key and its posterior covering by soft tissues. (**h**–**j**) Clinical aspect of the dental implant after reopening and coupling the transmucosal healing screw. (**k**–**l**) Installation of the cotton thread in the peri-implant sulcus. (**m**) accumulation of biofilm on the cotton thread around the dental implant after five weeks of its installation.

At day 0, the animals were anesthetized using ketamine chloride (80 mg/Kg, Francotar, Virbac, SP, Brazil) and xylazine chloride (10 mg/kg, Rompum, Bayer, RS, Brazil) and the upper right incisors were removed (Fig. 5a). Antisepsis with 1% polyvidone-iodine followed by intraoral antisepsis with 0.12% chlorhexidine gluconate were performed. Minimally traumatic exodontia was performed, using adapted dental devices for the syndesmotomy of the soft tissues and careful mesiodistally and bucco-palatal luxation of the tooth with interfix and inter-resistant lever movements. A clinical tweezer was used to hold the tooth (Fig. 5b) and a bucco-palatal rotated traction movement was made the exodontia (Fig. 5c and d).

**Figure 6:**
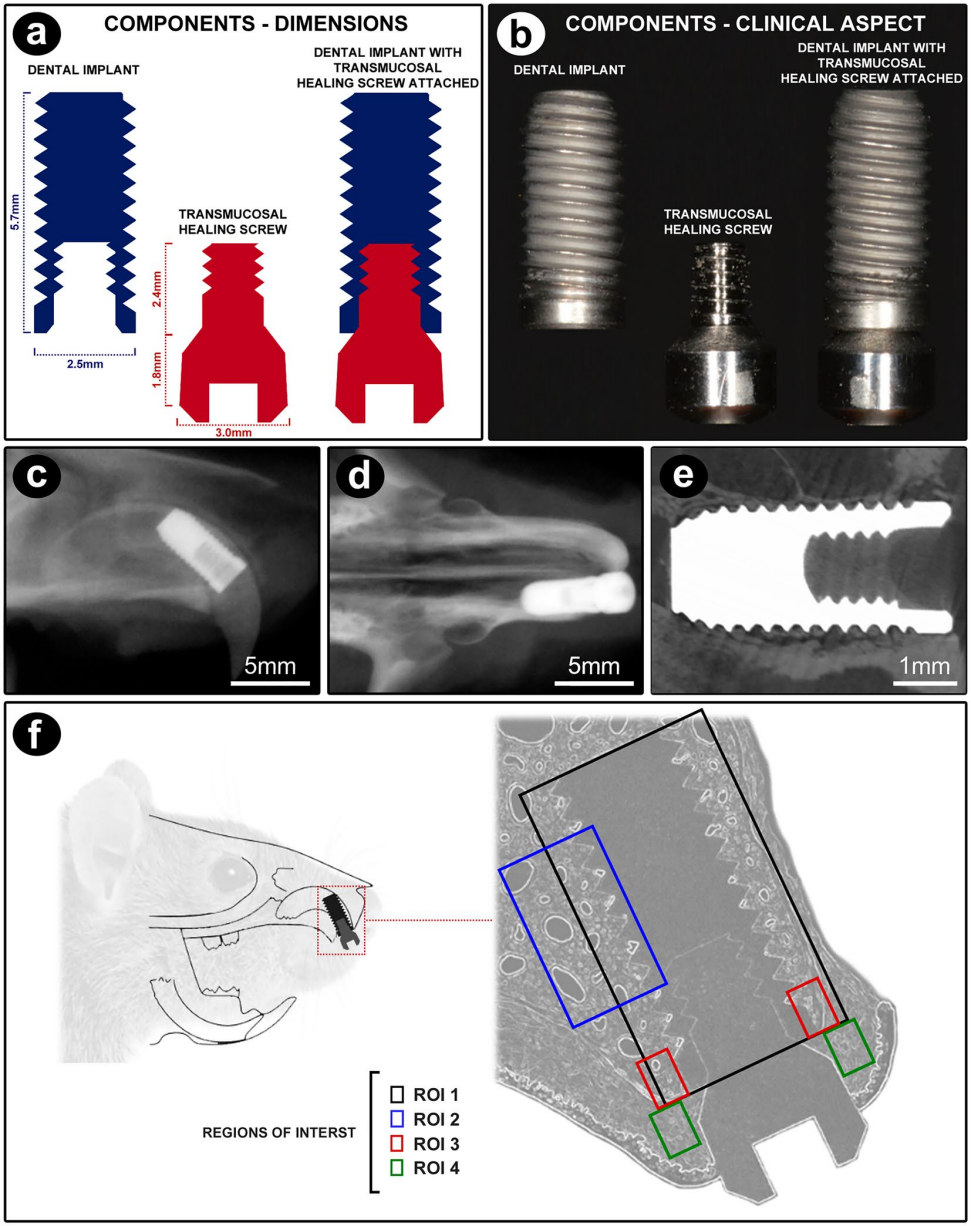
Dental implant and transmucosal healing screw characteristics and regions of interest (ROIs) evaluated in the present study. (**a**) Schematic drawing of the structure and dimensions of the dental implant and transmucosal healing screw. (**b**) Photograph showing the dental implant and the transmucosal healing screw separately and coupled to each other. Radiographic (**c**,**d**) and microtomographic (**e**) aspect of the dental implant after eight weeks of its installation in the maxilla (osseointegrated implant). (**f**) Schematic drawing showing the position of the dental implant in the maxilla of the rat and representation showing the ROIs analyzed in this study. In ROI1 the histopathological and PTBT analyzes were performed (black rectangle), in ROI2 the PNVBT analysis was performed (blue rectangles), in ROI3 the analyzes of OCN and TRAP were performed (red rectangle) and in ROI4 the analyzes of TNFα, IL-1β, VEGF (green rectangle) were performed.

A titanium DI (Dentfix, SP, Brazil) was especially designed for this, presenting 2.5 mm of diameter and 5.7 mm of length, and surface treated with blasting and acid etching (Fig. 6a,b). The DI was inserted using a 1.2 hexagonal digital key coupled to the internal connection of the implant (Fig. 5e,f), manually screwed until an adequate locking was achieved, and consequently, its primary stabilization by the side walls and curvature of the dental socket (Fig. 6c-e). Implant insertion was made in a slow and gradual way, respecting bone resilience, until the level of subgingival position.

After installation, peri-implant soft tissues were properly repositioned and sutured with 4.0 silk thread (Fig. 5g). Immediately after the surgery, all the animals were medicated with 0.01 mL/100 g of pentabiotics (Zoetis, SP, Brazil) via IM, in order to prevent eventual infectious conditions.

### Implant platform exposure

At week 8, the animals were anesthetized as mentioned previously and the implant platform was exposed (Fig. 5a). Local anesthesia with 2% lidocaine + 1:100.000 epinephrine (Alphacaine 100—New DFL, RJ, Brazil) was also performed, especially to reduce bleeding. A linear incision using a 15c scalp was made, perpendicular to the contralateral tooth on the implanted site, and a full thickness flap was folded (Fig. 5h). A 1.8 mm height transmucosal healing screw (Dentfix, SP, Brazil) was placed using a 0.9 hexagonal digital key (Fig. 5i,j) and the secondary stability of the implant was clinically checked and the surrounding tissues were sutured with a 4.0 silk thread.

### Treatment with vehicle or zoledronate

At week 9, treatment with vehicle or zoledronate was initiated, continuing until week 19. This protocol consisted in the intraperitoneal (IP) administration of 0.45 ml of vehicle (0.9% NaCl), or 0.45 ml of zoledronate (Sigma-Aldrich, MO, USA) (Fig. 5a). ZOL dosage (100 µg/Kg) was based in previous studies^54,68–70^ and was adapted from the oncologic therapy used in humans for the rats.

### Experimental groups

At week 9, the animals were randomly distributed in four groups, according to the treatment: VEH group (n = 7)—treated with vehicle, VEH-EPI group (n = 7)—treated with vehicle and with ligature-induced experimental peri-implantitis, ZOL group (n = 7)—treated with zoledronate, and ZOL-EPI group (n = 7)—treated with zoledronate and with ligature-induced experimental peri-implantitis (Fig. 5a).

### Experimental peri-implantitis

At week 14, the animals from VEH-EPI and ZOL-EPI groups were anesthetized as mentioned previously and a cotton ligature was installed (#24; Coats Corrente Ltda., Brazil) around the cervical portion of the transmucosal healing screw (Fig. 5a), in order that the ligature was kept in position in the peri-implant sulcus (Fig. 5k,l) causing microbial biofilm accumulation, and consequently, leading to EPI (Fig. 5m). The ligature was left in position until the end of the experimental period, at week 19 (Fig. 5a).

### Post-operatory care

During all the experimental period after DI installation, the animals received crushed food to facilitate feeding and to minimize biomechanical stress on the DI. Considering that rodents present a continuing eruption of the incisors, the lower incisors were weekly sanded to enable feeding and avoid injury to the implanted site.

### Euthanasia

At week 19, the animals were deeply anesthetized before undergone transcardiac perfusion with 100 ml of 0.9% NaCl solution with 0.1% of heparin, followed by 800 ml of 4% of formaldehyde (Sigma-Aldrich) in 0.1 M phosphate-saline buffer (PBS) at 4 °C and pH 7.4.

### Histological processing

Maxillae containing the osseointegrated DI were carefully dissected, post-fixated for 48 h and demineralized for 75 days in PBS with 10% ethylene-diaminetetraacetid acid (EDTA) (Sigma-Aldrich).

After demineralization, the implants were carefully removed with a 0.9 hexagonal digital key coupled with the internal connection of the transmucosal healing screw. The specimens were dehydrated, diaphanized, and paraffin embedded. Histological slices of 4 μm thickness were obtained following the long axis of the DI, from medial to distal. Histological sections of the central region of the implant site were collected and placed in silanized slides. Part of the samples were stained with hematoxylin and eosin (HE) for histological and histometric analysis, and part was used for immunohistochemistry.

### Immunohistochemistry processing

The histological sections were deparaffinized and rehydrated. Washes in PBS were repeated at the end of each immunohistochemistry reaction step. For antigenic retrieval, the slices were immersed in citrate buffer in pressurized chamber (Decloaking chamber, Biocare Medical, CA, USA). For the endogenous peroxidase blockage, the histological sections were treated with 3% of hydrogen peroxide (Sigma-Aldrich) for 1 h. Nonspecific sites were blockaded with 1.5% bovine albumin serum (BSA) (Sigma-Aldrich) for 12 h.

The histological slices were divided in five batches, and each one was incubated with one of the following primary antibodies: rabbit alfa tumoral necrosis factor (TNFα) antibody (orb11495, 1:100; Biorbyt, Cambridge, UK); rabbit interleukin 1-beta (IL1-β) antibody (orb382131, 1:100; Biorbyt); rabbit vascular endothelial growth factor (VEGF) antibody (orb191500, 1:100; Biorbyt); mouse osteocalcin (OCN) antidody (sc365797, 1:100; Santa Cruz Biotechnology, TX, USA) and mouse tartrate resistant acid phosphatase (TRAP) antibody (sc376875, 1:200; Santa Cruz Biotechnology). After 24 h of incubation in the primary antibodies, a second incubation with a biotinylated horse anti-mouse/rabbit IgG antibody (BA-1400, 1:100; Vector Laboratories, CA, USA) was made for 1 h, followed by an incubation with streptavidin-conjugated with HRP (SA-5004, 1:100; Vector Laboratories). Immunoperoxidase was revealed with 3,3’- diaminobenzidine (SK-4100, ImmPACT DAB Substrate kit, peroxidase, Vector Laboratories). No counter staining was made in the slices immunolabed with TNFα, IL1-β, VEGF and OCN. TRAP slices were counterstained with Harris hematoxylin for 5 min. For negative controls, the primary antibodies were omitted.

### Analysis of the results

#### Analysis of the general health of the animals and intraoral condition

During the experimental period, general health of the animals was monitored, and eventual intercurrence were registered and analyzed. Body weight was registered at three distinct moments during the experiment, before the exodontia and DI installation, before platform exposition, and before euthanasia. Results were represented in grams under mean ± standard deviation for each group. Intraoral exam was performed by means of a careful inspection of the region to be operated or of the peri-implant site and surrounding tissues.

#### Microscopical analysis and regions of interest (ROI)

Microscopic analysis was performed by a calibrated examiner (E.Q.M.S.) and validated by a certified histologist (E.E.), both blinded to the experimental groups, using a light microscope (Axio Scope, Carl Zeiss Microscopy GmbH, NI, Germany) with a digital camera (AxioCam MRc5, Carl Zeiss Microscopy GmbH, NI, Germany) connected to the computer. The photomicrographs of the histological sections were captured obeying the different ROIs related to the microscopical analysis (Fig. 6f), using ZEN2 (Carl Zeiss Microscopy GmbH, NI, Germany).

Four distinct ROIs were determined, named ROI-1, ROI-2, ROI-3 and ROI-4. The dimensions and the positioning of each ROI represented in Fig. 6f. ROI-1 was defined as a 6264 μm x 4716 μm rectangle, with the longer side corresponding to the long axis of the implant including all the peri-implant tissues in all the entire length of the implant. ROI-2 was a 2784 μm x 2096 μm rectangle, with the longer side corresponding to the long axis of the implant. This rectangle was placed in the palatal side of the implant, including their central threads, in a way that in its proximal half it included the threads and adjacent peri-implant tissues, those newly formed during tissue healing and osseointegration, while its distal half included the pre-existing bone tissue. ROI-3 was represented as two rectangles of com 348 μm x 262 μm, with the longer sides accompanying the long axis of the implant. These rectangles were placed in the peri-implant bone tissue of the palatal and buccal sides of the implant, respectively, positioned in the center of the alveolar process, with the bone crest marking the coronal limit. ROI-4 was also represented as two 348 μm × 262 μm rectangles, with the longer sides accompanying the long axis of the implant, placed in the peri-implant mucosa of the palatal and buccal sides of the implant, respectively, with its central portion occupied by the peri-implant supra-alveolar connective tissue. The different ROIs were determined according to the type of microscopical analysis to be performed, in order to offer representative and trustworthy to the cell and tissue conditions of the peri-implant region.

### Histopathological analysis

Histopathological analysis was made in ROI-1, where the following parameters were evaluated: *a.* cell pattern and peri-implant soft tissues structure, with special attention to the peri-implant connective tissue; *b.* cell pattern and peri-implant bone tissue structure; *c.* presence, nature and intensity of local inflammation; *d.* the extension of inflammation, if present.

#### Histometric analysis of the percentage of total bone tissue (PTBT) and non-vital bone tissue (PNVBT)

Histometry was made in order to quantify the PTBT and PNVBT in peri-implant region. The photomicrographs previously acquired from ROI-1 and ROI-2 were analyzed using ImageJ® (National Institute of Health, MD, USA) software, and the respective measurements were made using *Polygon Selections* tool. For PTBT, the total bone area in ROI-1 was first measured and the area correspondent to the space previously occupied by the implant was subtracted, and the result considered as 100%. Thereafter, only the area occupied by bone tissue inside the ROI-1 was measured, to which it was assigned the correspondent percentual tax. For PNVBT, the total bone area inside ROI-2 was first measured and defined as 100%. In the sequence, only the area correspondent to non-vital bone tissue in this region, characterized by the presence of contiguous lacunae with no osteocytes and/or filled by osteocytes’ remnants, to which it was assigned the correspondent percentual tax. The results were expressed in percentage (%) under mean ± standard deviation for each group.

#### Immunohistochemistry for TNFα, IL-1β, VEGF, OCN and TRAP

For analysis of immunohistochemistry for TNFα, IL-1β and VEGF, ROI-4 was used. For OCN, ROI-3 was the region of analysis. Using ImageJ^®^ software, the immunolabeling was demarcated using *Color Threshold* tool in order to obtain the density of the immunolabeling in each ROI. The results were expressed in percentage (%) under mean ± standard deviation for each group^69^. ROI-3 was also used for TRAP analysis using ImageJ^®^, and TRAP-positive cells were quantified. The results were expressed considering the number of cells per mm^2^, under mean ± standard deviation for each group.

### Statistical analysis

In the present study, PNVBT was considered as the primary outcome. Histopathological analysis, PTBT and immunolabeling for TNFα, IL-1β, VEGF, OCN and TRAP were considered secondary outcomes.

For statistical analyzes the Bioestat 5.3 (https://www.mamiraua.org.br/pt-br/downloads/programas/bioestat-versao-53; Mamiruá Institute, AM, Brazil) software was used.

Sample size determination was based on a pilot study carried out by our research group. The sample size was calculated to ensure a power greater than 80% (α of 5%; type B error of 20%). It was determined that 5 would be the minimum number of repetitions required per treatment. Therefore, so that complications throughout the experimental period would not compromise the study, a number of 7 animals was established in each of the experimental groups.

The Shapiro–Wilk test was used to analyze the distribution of data referring to PTBT, PNVBT and immunolabeling for TNFα, IL-1β, VEGF, OCN and TRAP. Considering that all variables evaluated present a normal distribution, Analysis of variance (ANOVA) was used followed by Tukey post-test, considering the level of significance of 5% (p < 0.05).

### Supplementary Information

Supplementary Figures.

## Data Availability

The datasets used and/or analysed during the current study available from the corresponding author on reasonable request.
