# Supplementary material for: Peri-implantitis increases the risk of medication-related osteonecrosis of the jaws associated with osseointegrated implants in rats treated with zoledronate
Source: Sci Rep. 2024 Jan 5;14:627. doi: 10.1038/s41598-023-49647-4 (PMC10770413; doi:10.1038/s41598-023-49647-4)
Supplement: Supplementary file 1 — Supplementary Figures. [file 41598_2023_49647_MOESM1_ESM.pdf]

SUPPLEMENTARY MATERIAL

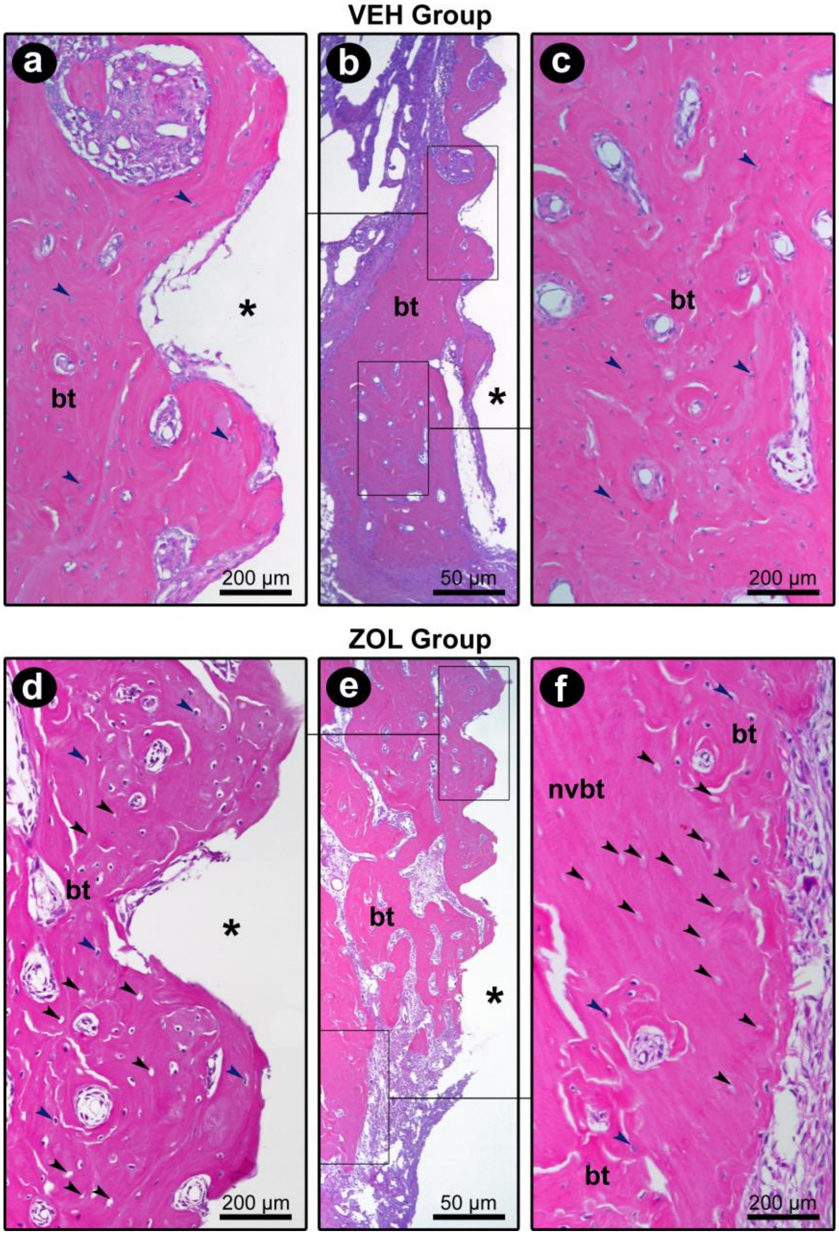

Supplementary material 1. Histological appearance of the peri-implant tissues in the VEH **(a – c)** and ZOL **(d – f)** groups. In **(b)** and **(e)** photomicrographs at lower magnification in which it is possible to observe the site previously occupied by the dental implant (asterisks) and the tissues located around it. In **(a)** and **(d)** it can be see the bone tissue filling the dental implant threads and in **(c)** and **(f)** the appearance of the bone tissue located adjacent to it. Note the large amount of non-vital bone tissue in the ZOL group. Abbreviations and Symbols: asterisks, site previously occupied by the dental implant; bt, bone tissue; nvbt, non-vital bone tissue; blue arrows, osteocytes; black arrows, empty lacuna. Original magnification: **a, c, d, f:** 200x; **b, e:** 50x. Scale bars: **a, c, d, f:** 200µm; **b, e:** 50µm.

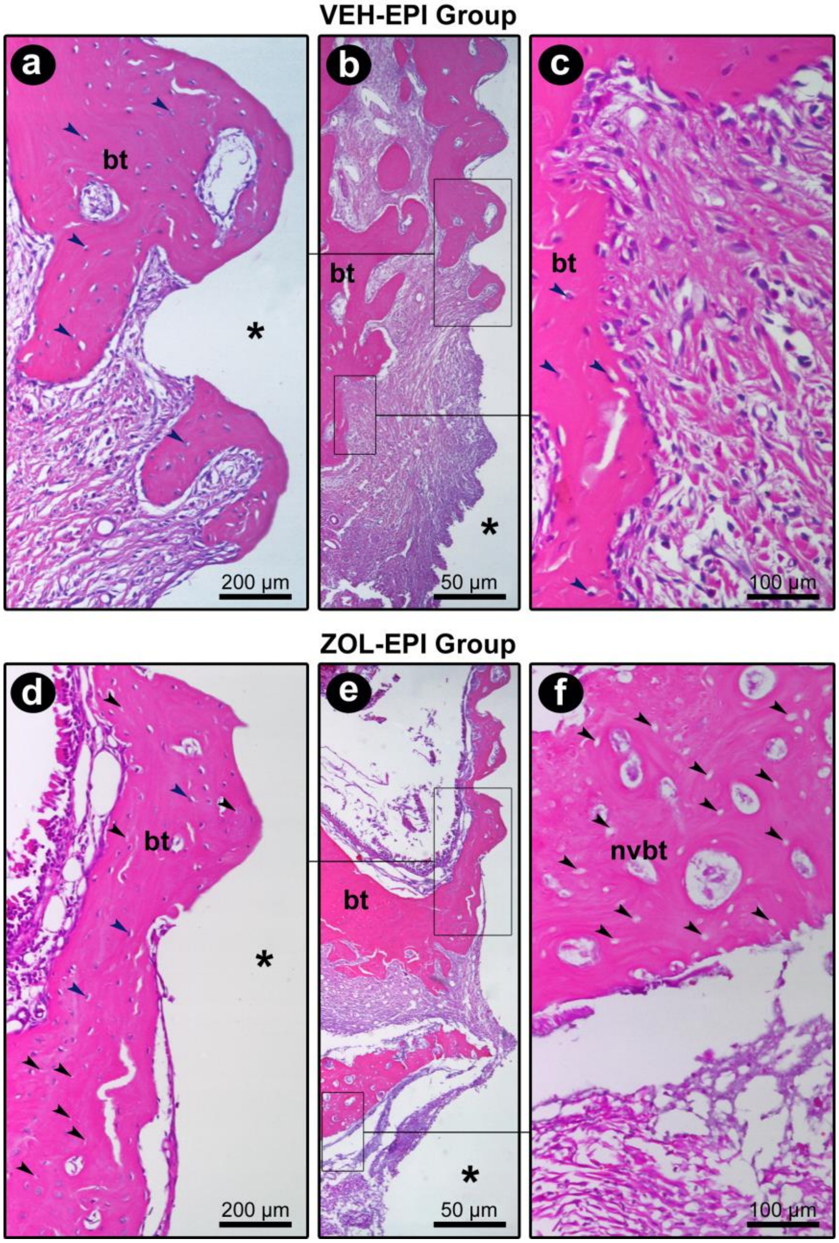

Supplementary material 2. Histological appearance of the peri-implant tissues in the VEH-EPI **(a – c)** and ZOL-EPI **(d – f)** groups. In **(b)** and **(e)** photomicrographs at lower magnification in which it is possible to observe the site previously occupied by the dental implant (asterisks) and the tissues located around it. In **(a)** and **(d)** it can be see the bone tissue filling the dental implant threads and in **(c)** and **(f)** the appearance of the bone tissue located adjacent to it. Note the bone loss in VEI-EPI group **(b)** and ZOL-EPI group **(e)**, which is more pronounced in this last. Note in VEI-EPI group **(b)** and ZOL-EPI group **(e)** the intense inflammatory infiltrate in the peri-implant soft tissues, and in this last there is a severe disruption of such tissues. Note the large amount of non-vital bone tissue in ZOL-EPI group **(e, f)**. Abbreviations and Symbols: asterisks, site previously occupied by the dental implant; bt, bone tissue; nvbt, non-vital bone tissue; blue arrows, osteocytes; black arrows, empty lacuna. Original magnification: **a, d:** 100x; **b, e:** 50x; **b, e:** 200x. Scale bars: **a, d:** 200µm; **b, e:** 50µm; **c, f:** 100µm.
